# Polyamide as a Denture Base Material: A Literature Review

**Published:** 2015-03

**Authors:** Mahroo Vojdani, Rashin Giti

**Affiliations:** 1 Biomaterial Research Center, School of Dentistry, Shiraz University of Medical Sciences, Shiraz, Iran;; 2 Postgraduate Student, Dept. of Prosthodontics, School of Dentistry, Shiraz University of Medical Sciences, Shiraz, Iran;

**Keywords:** Polyamide, Denture base, Nylon

## Abstract

The purpose of this article was to review the biocompatibility, physical, and mechanical properties of the polyamide denture base materials. An electronic search of scientific papers from 1990-2014 was carried out using PubMed, Scopus and Wiley Inter Science engines using the search terms “nylon denture base” and “polyamide denture base”. Searching the key words yielded a total of 82 articles. By application of inclusion criteria, the obtained results were further reduced to 24 citations recruited in this review. Several studies have evaluated various properties of polyamide (nylon) denture base materials. According to the results of the studies, currently, thermo-injectable, high impact, flexible or semi-flexible polyamide is thought to be an alternative to the conventional acrylic resins due to its esthetic and functional characteristics and physicochemical qualities.

It would be justifiable to use this material for denture fabrication in some cases such as severe soft/ hard tissue undercuts, unexplained repeated fracture of denture, in aesthetic-concerned patients, those who have allergy to other denture base materials, and in patients with microstomia.

Although polyamide has some attractive advantages, they require modifications to produce consistently better properties than the current polymethyl methacrylate (PMMA) materials. Moreover, since there is a very limited knowledge about their clinical performance, strict and careful follow-up evaluation of the patients rehabilitated with polyamide prosthesis is recommended.

## Introduction

Polymethyl methacrylate (PMMA) has been the most popular material used for denture fabrication since its introduction in 1937.[1] It has several advantages such as an excellent esthetic characteristic, low water sorption and solubility, adequate strength, low toxicity, easy repair, and a simple molding processing technique. Nonetheless, it has some problems such as polymerization shrinkage, weak flexural, lower impact strength, and low fatigue resistance.[1-4] These often lead to denture failure during chewing or when fall out of the patient’s hand. In order to enhance some properties of PMMA, various efforts have been taken including addition of metal wires or plates, fibers,[5-8] metal inserts,[9] and modification of chemical structure. In recent years, nylon polymer has attracted attention as a denture base material. Polyamide resin was proposed as a denture base material in the 1950s.[10] Nylon is a generic name for certain types of thermoplastic polymers belonging to the class known as polyamides. These polyamides are produced by the condensation reactions between a diamine NH_2_-(CH_2_)_6_-NH_2_ and a dibasic acid, CO2­H-(CH­_2_)_4_-COOH.[11-15] Nylon is a crystalline polymer, whereas PMMA is amorphous. This crystalline effect accounts for the lack of solubility of nylon in solvents, as well as high heat resistance and high strength coupled with ductility.[16-17] Moreover, it was claimed that nylon materials have other advantages including higher elasticity than common heat-polymerizing resins,[12] toxicological safety for patients with resin monomer and metal allergy,[18] use of heat-molding instead of chemical polymerization to control the polymerization shrinkage and its related deformation.[18] On the other side, it is reported that this material has several problems such as water sorption, surface roughness, bacterial contamination, warpage, color deterioration, and difficulty in polishing.[19] The present study is a literature review to appraise some physical, mechanical and clinical properties of nylon/ polyamide denture base materials.

## Method

**Figure 1:**
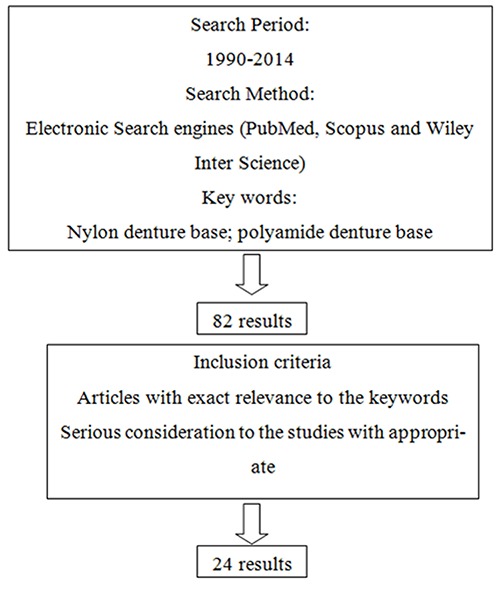
Method of searching and selecting the articles

This study is a structured literature review of articles published from 1990 to 2014. PubMed, Scopus, and Wiley Inter Science databases were used to search “nylon denture base” and “polyamide denture base” key words. The search was limited to English language publications. The articles were reviewed by two experts in the field of prosthodontics. Searching the key words yielded a total of 82 articles. As the inclusion criteria, the publications had to be exactly related to the key words; no editorials and manufacturer-supported publications were accepted for review process. By application of inclusion criteria, the obtained results further reduced to 24 citations that formed the basis for this review (Figure 1).

*Flexural Properties*

There are some studies that have evaluated the mechanical properties like flexural strength, modulus of elasticity, deflection at breakage, and tensile strength of nylon as a denture base material.

Yunus *et al.* in 2005 [19] evaluated some flexural properties of a nylon denture base material (Lucitone FRS) compared with a conventional compression-molded heat-polymerized (Meliodent), a compression-molded microwave-polymerized (Acron MC) and an injection-molded microwave-polymerized (Lucitone 199) PMMA polymers. They found nylon to have the lowest flexural modulus of 1714 MPa when not disinfected, while the disinfected specimens (with an oxygen- releasing disinfectant solution had a higher value of 1937 MPa. In this study, nylon showed a lower flexural strength than the two compression-molded PMMA polymers but a comparable value with Lucitone 199.

Takabayashi in 2010 [20] compared some characteristics of six thermoplastic denture resin materials (three polyamide, two polycarbonate, and a polyethylene terephthalate resin). In this study, flexural strength and modulus of elasticity of polyamide type materials (Val-plast, Lucitone FRS and Flexite Supreme) were lower than what was required according to the ISO standard. However, they demonstrated great toughness and strong resistance to fracture in comparison with conventional polymethyl methacrylate (Acron). Also the tensile strength test showed that polyamide can withstand stress through a considerable degree of deflection. This characteristic submitted the advantage for non-metal clasp dentures because of providing retention through the use of undercut on the remaining teeth, and thus alleviating the denture pain caused by the excessive local pressure.

Hamanaka *et al.* in 2011,[21] compared some mechanical properties of two polyamides (Nylon 12 and Nylon PACM12), one polyethylene terephthalate and one polycarbonate with a conventional heat-polymerized polymethyl methacrylate (PMMA). They showed that the two polyamides had the lowest values of flexural strength at proportional limit as well as the lowest elastic moduli between denture base resins. They also found that Charpy impact strength was the highest for Nylon PACM12, while Nylon 12 had low impact strength. This study demonstrated that the mechanical properties of injection-molded thermoplastic denture bases differ from each other; hence, the clinicians should be well aware of these properties in order to choose the most suitable one for an RPD without metal clasps that is suitable for each patient.

In 2012, Ucar *et al.*[22] evaluated some mechanical properties of a polyamide-based denture material (Deflex) and contrast it with another injection-molded PMMA base material and a conventional compression-molded PMMA. The results revealed that the polyamide flexural strength was not significantly different from compression-molded PMMA and that the flexural modulus of polyamide was lower than compression-molded PMMA material. The major connector of a removable denture should be rigid enough to evenly distribute the applied force on the denture. Therefore, it was emphasized that a lower flexural modulus (higher flexibility) has been often a disadvantage from clinical standpoint.[23-25] Another study in 2012[26] reported that thermocycling significantly decreased the flexural strength and elastic modulus of one polyamide (Valplast), while it significantly increased the same features in the other polyamide (Lucitone FRS). The impact strength of one of the polyamides (Lucitone FRS) also decreased by thermocycling, revealing that thermal stress would affect the mechanical properties of these materials. In the study performed by Soygun *et al.* in 2013,[27] mechanical and thermal properties of polyamide (Valplast) were compared with the conventional PMMA (control) and fiber reinforced PMMA denture base materials. The results of the transverse test were determined by the three-point flexure test on a computer-aided universal test device; it showed that the polyamide based denture base material (Valplast) had the highest transverse strength (117.22± 37.8 MPa), and no fracture was observed in these specimens. The values of modulus of elasticity in all experimental groups were lower than that of the control group. It was also observed that the values of maximum impact strength were the highest for polyamide and it was much higher than the other groups. This could be attributed to the chemical structure properties of Valplast which enables it for a better force absorption and is different from those of PMMA. In 2013, Wadachi *et al.*[28] compared the rigidity of dentures made of a polyamide resin (Valplast), a polyester resin (Esthe Shot) and a conventional heat-polymerized resin (Physio Resin). They showed that the denture made of polyamide resin had the lowest elasticity; therefore, it was reasonable to think that this material allowed for the most considerable displacement of denture and permitted the pressure to be applied under the denture base. They concluded that this material needed to be reinforced by using metal frames in order to prevent the deformations caused by occlusal forces.

*Water sorption and water solubility*

Lai *et al.* in 2003,[29] studied the color stability, stain resistance and water sorption of one silicon, one copolyamide, and two heat-cured acrylic resins as removable gingival flange materials. In that study, copolyamide (Flexite Supreme) had absorbed the greatest amount of water, whereas silicone showed the least water uptake after 56 days of water storage.

In the study carried out by Takabayashi in 2010,[20] water sorption of two of the tested polyamide materials (Valplast and Flexite Supreme) met the ISO standard (32 µg/mm[3]), but Lucitone FRS revealed the highest water sorption due to the greater degree of hydrophilic characteristics supported by the contact angle measurements. It is thought that the higher the amide group concentration, the greater the water sorption. Therefore, it has been suggested that the amide group concentration, in the polyamide type denture base materials, could be adjusted to a level as low as that in popular industrial materials such as nylon 6 or 66.[20] On the other hand, in another study which was done by Shah *et al.* in 2014, the sorption and solubility of heat-cured polymethyl methacrylate denture base resin and flexible denture base resin were compared and it was found that heat-cured PMMA had more sorption and solubility values than flexible (thermoplastic polyamide nylon) resin.[30] The study suggested that since the contact angle between the flexible resin and water was high with low surface free energy, their water repellency was also high, and these all resulted in lower water sorption values. Likewise, it was mentioned that there was a strong hydrogen bonding between amide groups and a reduction in attachment areas for water molecules; therefore, the amount of water sorption in flexible resin was lower than conventional PMMA. The higher residual monomer contents were mentioned as a cause for the higher solubility levels of PMMA.[30]

*Hardness*

Ucar *et al.* in 2012[22]compared the hardness of a polyamide based denture material (Deflex) with another injection-molded PMMA base material and a conventional compression-molded PMMA. The results of the study on Deflex specimens were found to be much lower than other materials and that material was not as hard as other materials. In the study by Shah *et al.* (2014)[30] PMMA demonstrated higher hardness values when compared with flexible resin. This result might be attributed to a high monomer-polymer ratio, the attachment of this material, and the presence of methyl-methacrylate monomer. Moreover, cross-linking agents may exist in the material. Flexible resin demonstrated lower hardness values and also possessed lower amounts of cross-linking agents, indicating that cross-linking agent may affect surface hardness.

*Color stability and stain resistance*

In the study of Lai *et al. *in 2003,[29] the color stability of 1 copolyamide (Flexite Supreme), 1 silicone (Gingivamoll) and 2 heat-polymerized acrylic resins (QC-20 and Vertex) as removable gingival flange materials were evaluated by a spectrophotometer after 7, 14, 30, 120, and 180 days of immersion in staining solutions of coffee and tea. Copolyamide had the greatest staining in tea solution, the silicone material in coffee solution. The color of all materials remained in air and water for 6 months, showing that extrinsic stains had played a major role in the discoloration of the materials in this study. The color changes of silicone and copolyamide materials stored in coffee solution for 180 days were greater than 3 NBS (National Bureau of Standards) units, which would be characterized considerable and deliberated clinically unacceptable. Takabayashi *et al.* in 2010[20] compared the color stability of six thermoplastic denture resin materials (three polyamides, two polycarbonates and a polyethylene terephthalate) after being soaked in coffee and curry solutions for 60 hours. In that study, three polyamides (Valplast, Lucitone FRS and Flexite) had considerable color change in the curry solution and Valplast and Flexite showed considerable color change after soaking in the coffee solution. Sepúlveda-Navarro *et al.* in 2011[31] compared the color stability of two heat-cured denture base acrylic resins (Lucitone 550, VipiCril) with the thermoplastic nylon resin (Transflex) in different beverages (coffee, Cola, red wine, and distilled water) by using an ultraviolet-visible spectrophotometer. The most severe staining was shown with red wine followed by coffee; Transflex showed a significant color change after 15 and 30 days of immersion in Cola. The larger color changes for nylon denture base materials would be related to their hygroscopic and also higher water sorption properties.[32-33] It was found that the frequency of amide groups along the chain had affected the water sorption and the chemical properties of each type of nylon.[29] Another attributed reason could be the differences in finishing and polishing of nylon materials compared to PMMA. Rougher surfaces are more susceptible to staining.[34-36]

*Bond strength to other materials*

Auto-polymerizing resin is often used as reline or repair material for PMMA denture base,[37] but there are very limited studies in regard to bonding of polyamides to auto-polymerizing resin materials.

Katsumata *et al.* in 2009[38] studied the shear bond strength of an auto-polymerizing resin to a nylon denture base polymer (Lucitone FRS) subjected to different surface treatments and compared it with a heat-polymerizing resin and a polycarbonate polymer. The surface treatment methods were alumina sandblasting, resin primer coating, alumina sandblasting plus resin primer coating, and silica coating with Rocatec system. They also evaluated the effect of thermal cycling in this property. For the nylon polymer specimens, thermal cycling significantly reduced the bond strength of all groups except for the groups of silica coating with Rocatec system, in which no significant difference was found between thermal cycled and non-thermal-cycled specimens. This result clearly showed that the silica coating could improve the bonding strength of nylon denture base polymer to auto-polymerizing resin used for repairing and adjustment of nylon dentures. Nylon is a chemical-resistant material due to its high crystalline characteristics.[39] This property is opposite to PMMA denture base materials. Vojdani *et al.* showed that bond strength of repair materials increased significantly after chemical treatments of denture base materials.[40] However for nylon polymer, it is hard to react with the chemical etchant and primers of auto-polymerizing repair resins. Therefore, enriched bonding could not be achieved when nylon material is treated by polishing, alumina sandblasting and resin primer.

In another study in 2013,[41] it was demonstrated

that tribochemical silica coating and 4-META/MMA-TBB resin could cause the greatest post-thermocycling bond strength to polyamides (Valplast, Lucitone FRS) among the different surface treatment methods used in this study (air abrasion with alumina, dichloromethane, ethyl acetate, 4-META/MMA-TBB resin, alumina and 4-META/MMA-TBB resin, tribochemical silica coating, and finally tribochemical silica coating and 4-META/MMA-TBB resin). 

Polyamide was exceedingly difficult to bond to an auto-polymerizing repair resin; also the shear bond strength could improve using tribochemical silica coating followed by application of 4-META/MMA-TBB resin. Korkmaz *et al.* in 2013[42] used peel test to evaluate the bond strength of a silicon-based soft denture liner (Molloplast B) to PMMA and polyamide after laser application or air abrasion of denture resins. They showed that in the Deflex group (polyamide), the highest peel bond strength was observed when it was treated by Er, Cr: YSGG laser at 2W-20HZ, while the lowest peel bond strength was recorded in the group which was sandblasted with Al_2_O_3_. Therefore air abrasion of polyamide resins should be avoided in order not to impair their bond strength to silicon-based soft denture liners.

*Dimensional accuracy of nylon denture base materials*

Despite several advantages of PMMA, one of its main disadvantages is polymerization shrinkage during processing. For solving this problem, various injection-molded materials and processing techniques are now available.[43] The study of Stafford *et al.*[18] was the first attempt to study the dimensional accuracy of nylon denture-base materials. Parvizi *et al.* in 2004[44] compared the dimensional accuracy of an injection-molded nylon denture base material with one conventionally processed PMMA, one injection-molded PMMA, and an injection-molded styrene. They found that all of the materials exhibited some degree of shrinkage as a result of processing, but this shrinkage was highest for nylon with 2.5% in the cross arch dimension, which was 2.8 times greater than the conventionally processed PMMA. The smallest mean shrinkage was associated with styrene and the largest with nylon. The dimensional change of nylon was clinically significant, and could have an effect on the final fit of the denture. The lower dimensional accuracy of nylon was shown to be related to its technique sensitivity during the processing stages. Also its dimensional change could be affected by water sorption when considering nylon as a hydrophilic material.[44-45]

*Surface roughness*

A rougher surface can cause discomfort to patients and also discoloration of the prosthesis; it may contribute to microbial colonization and biofilm formation.

Abuzar *et al. *in 2010[46] evaluated the surface roughness of a polyamide denture base material (Flexiplast) in comparison with PMMA (Vertex RS), and found that polyamide specimens produced a rougher surface than PMMA, both before and after the polishing process. The unpolished polyamide surface might have been affected by some degrees of disintegration of the mold surface which was heated to a higher temperature compared to PMMA, and also the pressure during injection molding.[18] Similar to polymethacrylate resin materials,[47] the conventional polishing technique provided a polyamide surface smoothness, well within the clinically acceptable standard. The same results were found in another study done by Kawara *et al.* in 2014[48]who evaluated the surface roughness of four thermoplastic (polyamide: Valplast, Lucitone FRS, polyethylene terephthalate: EstheShot, and polyester: EstheShot Bright) and two conventional acrylic (Heat-polymerizing: Urban, and Pour type auto-polymerizing: Pro-Cast DSP) denture bases by using scratch test. The results showed that the surface of thermoplastic denture base resins was easily damaged compared with polymethyl methacrylate.

*Effect of denture cleansers on polyamide denture base materials*

Adhesion of microorganisms, especially yeasts, to the denture base materials is an important issue that compromises its service and efficacy.[49] Although too many researches have been conducted to control the development of pathogenic biofilm on PMMA materials;[50-52] the studies concerning the effect of disinfecting methods on polyamides are very limited. In 2011,[53] a study was conducted on the effect of denture cleansers on the formation of Candida biofilms on a polyamide resin (Flexite M.P) and a polymethyl methacrylate resin (Acron MC). The study showed that Candida biofilms had significantly higher growth on polyamide compared with PMMA indicating that polyamide could present a convenient surface for microbial colonization. These differences would be attributed to the higher amount of residual monomers in PMMA which produced differences in the resin surface-charge, being capable of reducing adhesion and inhibiting the growth of Candida. The results also showed that the denture cleanser solutions, with or without enzymes, were effective in controlling candida albicans biofilm levels in both polyamide and PMMA resins. In a study performed by Durkan *et al.* in 2013,[54] the effects of three sodium perborate-containing denture cleansers (CO-Corega, PR-Protefix, VA-Valclean) were evaluated on the surface roughness, hardness, and color stability of two polyamides (Valplast and Deflex), a butadien styrene copolymer PMMA (Rodex), and a PMMA polymer as a control group (Paladent). Surface roughness of the polyamide increased after 20 days of repeated immersion regardless of the type of the solution used. Valplast had a higher initial surface roughness which increased after the immersion. Polyamide resins demonstrated low Vickers hardness before and after immersion in the denture cleansers. Hardness of the polyamide resins and PMMA resin decreased after repeated immersions, regardless of the solution type. This study also showed that no changes occurred in the color of polyamides in these solutions that could be related to the short duration of immersion (20 days). Another study in 2014 evaluated the effects of two denture cleansing methods [Val-Clean (peroxide cleanser) and Corega Extradent (peroxide cleanser) plus microwaving] on 3-D surface roughness, gloss and color of nylon (Valplast), and heat-polymerized acrylic denture base material (Paladon 65) for a period simulating 30 days of daily cleansing, by using an interferometric profilometer, a gloss meter, and a colorimeter. The results of this study showed that cleansing methods had no different effect on color group compared with the control group, when using the same material. However, the effect on Valplast was higher than Paladon 65, both at a clinically perceptible level. The Val-Clean method was the only method that had no particular influence on the gloss of both tested materials. Corega Extradent plus microwaves significantly decreased the gloss of both materials. Surface roughness was affected significantly only by Corega Extradent plus microwaves and only for the Paladon 65 material. The color change (as an effect of cleansing agent) was not associated with gloss or surface roughness in any of the materials. However, gloss and surface roughness were highly associated in Paladon 65 and could be used for the prediction of each other.[55]

*Cytotoxic evaluation of polyamide*

There are several studies in regard to cytotoxicity of denture base materials.[56-59] It has been reported that the acrylic resins used for the fabrication of denture bases have displayed various degrees of in vitro cytotoxicity and in vivo allergic responses, which have been probably caused by non-reacting components that remain after the polymerization process.[60] Nevertheless, studies about cytotoxic effect of polyamides are very limited. Uzun *et al.*[61] investigated the long-term cytotoxic response of an injection-molded polyamide (Deflex) and heat- and cold-cured PMMA resins. According to the results of their study, all materials had a similar toxic effect in the short term and all tested materials reached the highest levels of toxicity after 8 weeks of their aging time. In their study, polyamide specimens had a comparable toxicity profile with the conventional PMMA denture base materials.

## Case reports

In 2013,[62] a clinical report described a combination of a nylon partial removable prosthesis and a traditional partial removable dental prosthesis for a Kennedy class II, modification 1, partially edentulous mandible. After two years, this combination was functioning well, although the nylon material surface showed some discoloration.

Sinch *et al.* in 2013[63] reported a case of maxillary and mandibular arch reconstruction with a nylon denture base material due to the aesthetic concerns of the patient. They concluded that flexible partial dentures (FPD) could be a good option for replacement of missing teeth when the patient is concerned about aesthetics. FPD had given an option of thinking beyond complex designing of cast partial dentures. They could be considered for treating any patients who need replacement of missing teeth with aesthetic concerns; however, proper care of the prosthesis must be taken in order to minimize the staining of the prosthesis, which otherwise affects the aesthetics of the prosthesis.

## Conclusion

Physical and clinical properties of polyamides were briefly discussed in this review article. Although the flexural strength and modulus of elasticity and rigidity of nylon (polyamide) denture base materials are relatively low, they demonstrate great impact strength, toughness, and resistance to fracture. It was suggested that by adding glass fibers to polyamides, their stiffness and other mechanical properties could be increased. The use of these materials for non-metal clasp dentures has some advantages regarding their esthetic and degree of retention. However, these materials show some degree of color instability in different beverages. The bond strength of these materials to the repairing resins is low, but it can be significantly enhanced by silica coating with Rocatec system. Using the denture cleansers would increase the surface roughness of these materials and their cytotoxicity increases after long-term use. It was demonstrated that polyamides have rougher surface than other resin materials, and it causes more bacterial and fungal colonization. Therefore, selection of thermoplastic resin and designs adaptation for each clinical case must be achieved only after complete indulgence of the properties of polyamide materials. 
